# Notices

**Published:** 1970-01

**Authors:** 


### APPOINTMENT VACANT

**ROYAL NAVAL RESERVE, SEVERN DIVISION.** A vacancy will occur this year for a Medical Officer in the permanent R.N.R. Applicants should be under 35 years of age and live within 25 miles of Bristol and preferably be in established practice. Previous Service experience not necessary. Preference will be given to a General Practitioner. Full details from the Senior Medical Officer, H.M.S. Flying Fox, Mardyke Wharf, Bristol, 8.

